# Supplementary material for: Predicting visual function by interpreting a neuronal wiring diagram
Source: Nature. 2024 Oct 2;634(8032):113–23. doi: 10.1038/s41586-024-07953-5 (PMC11446822; doi:10.1038/s41586-024-07953-5)
Supplement: Supplementary file 6 — CRF and ERF predictions for individual TmY4 and TmY9 cells. Analogous to Supplementary Data 3, but for TmY target types. Shown are the top four monosynaptic pathways, the strongest pathway passing through each of the top ten intermediary types (ranking from Extended Data Fig. 7), and the trisynaptic pathway Tm1–TmY–Dm3–TmY (see the section entitled Prediction of spatial normalization). [file 41586_2024_7953_MOESM6_ESM.zip › DataS4/TmY9q/720575940639630406.pdf]

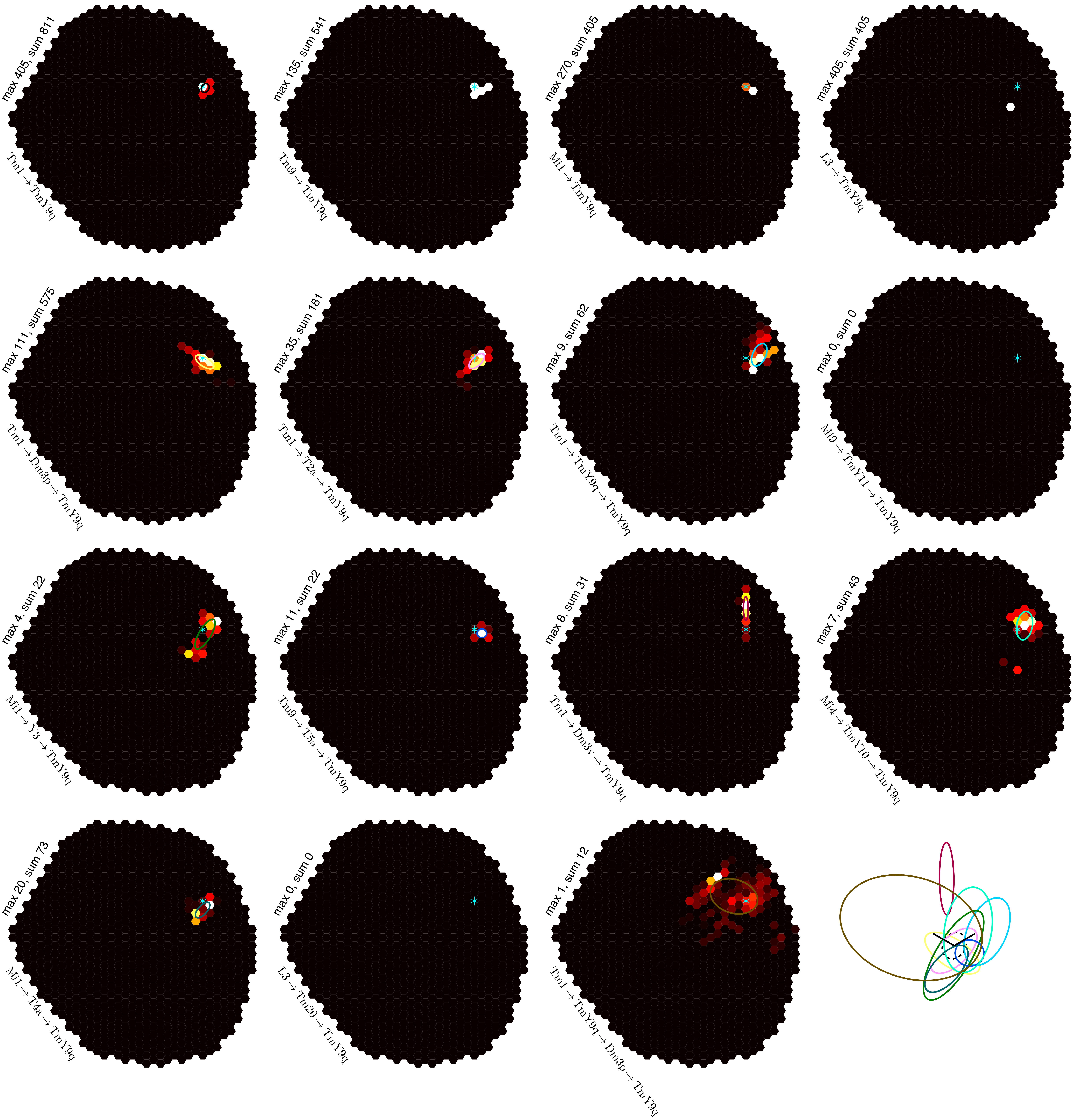
max 405, sum 811
Tm1 → TmY9q
max 135, sum 541
Tm9 → TmY9q
max 270, sum 405
Mi1 → TmY9q
max 405, sum 405
L3 → TmY9q
max 111, sum 575
Tm1 → Dm3p → TmY9q
max 35, sum 181
Tm1 → T2a → TmY9q
max 9, sum 62
Tm1 → TmY9q → TmY9q
max 0, sum 0
Mi9 → TmY11 → TmY9q
max 4, sum 22
Mi1 → Y3 → TmY9q
max 11, sum 22
Tm9 → T5a → TmY9q
max 8, sum 31
Tm1 → Dm3v → TmY9q
max 7, sum 43
Mi4 → TmY10 → TmY9q
max 20, sum 73
Mi1 → T4a → TmY9q
max 0, sum 0
L3 → Tm20 → TmY9q
max 1, sum 12
Tm1 → TmY9q → Dm3p → TmY9q
